# The preoperative temporal bone CT: a mnemonic for reducing surgical risk

**DOI:** 10.3389/fradi.2026.1784554

**Published:** 2026-05-20

**Authors:** Benjamin T. Ostrander, Vivian Vo, Adam Robinson, Jennifer Chang, Elina Kari

**Affiliations:** 1 Department of Otolaryngology – Head and Neck Surgery, University of California San Diego, La Jolla, CA, United States; 2 Department of Otolaryngology – Head and Neck Surgery, University of Minnesota, Minneapolis, MN, United States; 3 University of California San Diego School of Medicine, La Jolla, CA, United States; 4 Department of Radiology, University of California San Diego, La Jolla, CA, United States

**Keywords:** computed tomography, mastoid, neuroradiology, neurotology, temporal bone

## Abstract

Otologic surgery provides safe and effective treatment for a variety of ear and skull base pathologies. However, even routine procedures are not without risk of serious surgical complications. Ear surgery requires sub-millimeter tolerances near a variety of critical structures in a complex anatomic region. As such, preoperative computed tomography (CT) can be helpful to surgeons and radiologists for identifying anatomic variants that can influence surgical technique, and which may predispose patients to surgical complications. However, the analysis and reporting of temporal bone CT is not always detailed, consistent, or comprehensive. The purpose of this narrative review is to illustrate important landmarks and anatomic variants on the preoperative temporal bone CT, with special attention towards features important in otologic surgery and that may impact surgical risk. These critical landmarks and variants can be recalled using the mnemonic “**M**aking **M**iracles **I**nvolves **F**ocused **V**ision,” which provides an educational framework for the major regions that should be systematically reviewed in the imaging report: mastoid, middle ear, inner ear, facial nerve, and vascular anatomy.

## Introduction

The temporal bone, located at the base of the skull, plays a crucial role in hearing, balance, and overall function of the middle and inner ear. A comprehensive understanding of its anatomy is critical for the successful planning and execution of otologic surgery. Otologic procedures, such as mastoidectomy, cochlear implantation, and tympanoplasty, involve delicate manipulation of structures near the temporal bone's critical landmarks, including the facial nerve, the cochlea, the ossicular chain, and major vascular structures. Due to the complex anatomy and close proximity of these structures, any anatomic variant can significantly affect surgical technique as well as outcomes. Preoperative imaging, particularly computed tomography (CT), provides invaluable insight into the anatomy of the temporal bone and allows surgeons to identify potential risks that could complicate surgery. This narrative review explores a systematic approach to reading temporal bone CT pre-operatively and highlights the most significant landmarks and anatomical variants, emphasizing how these findings can influence surgical technique and outcomes.

## Overview of temporal bone anatomy

An intimate understanding of temporal bone anatomy is necessary to understand otologic pathology and is required for the safe and effective treatment of ear disease. The temporal bone has a pyramidal shape, the sides of which form the middle fossa floor superiorly, the anterior limit of the posterior fossa posteriorly, muscle attachments of the neck and intratemporal fossa anterior-inferiorly, and the musculocutaneous lateral border of the head which forms the base of the pyramid (1). Four embryologically distinct components comprise this bone: the squamous, mastoid, petrous, and tympanic parts (2).

The squamous part includes the zygomatic process, which forms the roof of the glenoid fossa and where the masseter muscle inserts. The temporalis muscle also inserts onto the squamous part, and forces from this muscle create a ridge of bone called the temporal line, which is used as a surface landmark to estimate the location of the middle fossa floor, on average approximately 5 mm superior to this line (3). The mastoid portion is a largely pneumatized bone shaped by the forces of the sternocleidomastoid and digastric muscles, which form the mastoid process inferiorly. The mastoid antrum is located deep to the cribriform area, posterior to the spine of Henle and inferior to the temporal line, an area known as MacEwen triangle (1). The petrous portion is the medial-most, pyramid-shaped bone that contains many critical structures. Surface features include the arcuate eminence, formed by the prominence of the superior semicircular canal. The petrous portion contains the labyrinthine and otic capsule bone, the hardest and densest bone in the body, and contains foramina and depressions for the passage of cranial nerves and great vessels. The tympanic portion is a ring-shaped bone that forms the bony external auditory canal. The tympanic membrane sits within this ring and is mechanically connected to the cochlea via the ossicular chain, namely the malleus, incus, and stapes, the smallest bone in the body.

The facial nerve follows a complex course from the brainstem through the temporal bone, with intratemporal segments including the labyrinthine, tympanic (horizontal), and mastoid (vertical) segments, before exiting the temporal bone at the stylomastoid foramen. A thorough understanding of the course of the facial nerve is essential for safe otologic surgery, and any variation in facial nerve anatomy must be reviewed prior to any surgery.

## Imaging modalities and protocols

The primary imaging modalities used to assess the temporal bone are computed tomography (CT) and magnetic resonance imaging (MRI). High-resolution CT offers excellent bony detail and is particularly useful in evaluating the mastoid, middle ear, and bony internal auditory canal (IAC) (4). CT is the gold standard for evaluating bony anatomy and is useful prior to performing stapededctomy, mastoidectomy, cochlear implantation, and some tympanoplasty and cholesteatoma surgeries. In contrast, MRI provides superior soft tissue contrast and is helpful for visualizing the fluid content of the labyrinth, the intratemporal portion of the facial nerve, the neural contents of the internal auditory canal, and skull base masses (5, 6).

The standard protocol for CT scans of the temporal bone typically involves a single axial acquisition that is reformatted into multiple planes, allowing comprehensive assessment of anatomical landmarks and potential variations (7). Conventional CT allows for the acquisition of images with slice thickness of 0.5 mm and slice interval of 1.5 mm, making it the most effective technique for visualizing small otologic structures. For patients presenting with specific symptoms, tailored imaging protocols are employed. For example, individuals with suspected otosclerosis often require high-resolution CT scans focused on the labyrinthine windows and cochlear capsules, ensuring the inclusion of the entire petrous temporal bone for thorough evaluation (8). Similarly, CT imaging plays a pivotal role in cochlear implantation planning, offering rapid acquisition and detailed visualization of osseous structures, which is essential for identifying surgical challenges such as cochlear malformation, anteriorly placed sigmoid sinuses, poorly pneumatized mastoid bones, aberrant facial nerve course, or narrow facial recess (9, 10). In superior semicircular canal dehiscence, CT can confirm an anatomical correlate to explain symptoms, and Stenver and Poschl plane reformatting can be useful for characterizing a dehiscence (11). Preoperative CT is particularly important for evaluating bony structures, and its detailed imaging allows for the assessment of potential complications or anatomical variants that could impact the surgical approach.

Photon counting CT is an emerging imaging technique that offers improved spatial resolution, decreased noise, and reduced radiation dose, allowing for slice thickness as thin as 0.2 mm.^14^ Although its use is still being explored in the clinical setting, temporal bone imaging with photon counting CT has shown promising potential for visualizing the microscopic structures within the ear and temporal bone (1, 12, 13).

## Common otologic surgeries involving the temporal bone

Most otologic surgery requires precise navigation around the temporal bone's complex structures. Commonly performed otologic procedures include mastoidectomy, cochlear implantation, tympanoplasty, and stapedectomy. These surgeries are unified by a requirement to safely create windows of access through the temporal bone, allowing manipulation and modification of pathology and structures of interest while preserving function. Mastoidectomy is often indicated for chronic ear disease including chronic otitis media or cholesteatoma, where removal of the mastoid air cells, cholesteatoma matrix, and granulation tissue may be necessary (14). Mastoidectomy is also a necessary step in cochlear implantation, with additional removal of bone at the facial recess providing access to the cochlea for cochlear implant electrode insertion (15). Middle ear surgery, such as stapedectomy, can also be guided by CT imaging (16). Preoperative imaging can assess middle ear pathology and further clarify causes of conductive hearing loss, allowing for analysis of ossicular chain integrity, otospongiosis, scarring or granulation tissue, middle ear opacification, modiolar anomalies, and third window defects. Tympanoplasty with or without ossicular chain reconstruction is performed for patients with tympanic membrane perforation and/or conductive hearing loss, often as a result of eustachian tube dysfunction or chronic otitis media (17). In these cases, preoperative CT helps evaluate the condition of the ossicular chain and the ventilation status of the middle ear. Additionally, lateral skull base tumors such as vestibular schwannoma, meningioma, and paraganglioma can be removed via a variety of craniotomy approaches, with the exact approach dictated in part by pre-operative imaging (18). Defects in the skull base, including encephaloceles and canal dehiscence, are carefully evaluated by imaging prior to attempting surgical repair. Systematic preoperative imaging review is essential for understanding each patient's anatomy prior to surgical intervention.

While CT imaging is not essential in every otologic procedure, we believe preoperative imaging is indicated in most ear surgery, including all cochlear implantation and the majority of middle ear and mastoid surgery. Imaging is useful in evaluating the extent of disease, identifying anatomic variants that narrow or obstruct access to critical structures, and assessing for anatomic variants that predispose patients to surgical complications (19). Imaging allows the best insight into all relevant anatomical details and potential situations which preclude surgery or require modifying standard surgical approaches (20).

## Preoperative imaging report

A detailed and systematic review of preoperative temporal bone CT is essential to ensure that all important structures and relevant anatomic variants are evaluated and documented. Using a mnemonic-based approach, this narrative review outlines key factors that should be considered when interpreting temporal bone CT scans. This systematic educational framework helps to standardize CT temporal bone review and ensure key anatomical features and variants are always evaluated. In 2016, O’Brien Sr et al. published the “CLOSE” algorithm for reviewing pre-operative sinus CTs (19). However, to our knowledge no such mnemonic exists for preoperative review of temporal bone CTs. We propose an educational framework using the mnemonic “**M**aking **M**iracles **I**nvolves **F**ocused **V**ision” as a tool for recalling the major regions that should be reviewed in the preoperative temporal bone CT: mastoid, middle ear, inner ear, facial nerve, and vascular structures. Of note, this mnemonic also aligns with the commonly taught “outside in” approach, where the most lateral/superficial structures of the ear and temporal bone are reviewed prior to the deeper regions. This specific phrase was selected with words that hint at the temporal bone category while also being memorable. This approach ensures that critical landmarks and variants are systematically assessed.

There remains great variability with regard to the content contained within preoperative temporal bone CT imaging reports. A standard report describing “normal” or typical anatomy provides a good starting point for interpreting temporal bone CTs. This baseline report at our institution typically reads as follows: “The external auditory canal is clear. No thickening of the tympanic membrane. The tympanic cavity and mastoid air cells are clear. The ossicles are aligned. The internal auditory canal, cochlea, vestibule, vestibular aqueduct have normal appearance. The semicircular canals are covered by bone. No irregularity of the tegmen, sigmoid sinus plate, facial nerve canal, or carotid canal. Temporomandibular joint is aligned.”

In the following section of this review a detailed assessment of temporal bone anatomy and surgical danger areas is illustrated using the “**M**aking **M**iracles **I**nvolves **F**ocused **V**ision” mnemonic. Information detailing the individual components of the mnemonic is presented in Table 1. Using a mnemonic-based approach, radiologists and otolaryngologists have a simple and efficient means of recalling which critical structures need to be evaluated and documented in the preoperative imaging report. Each component of the mnemonic is described in the imaging report, with pertinent positives and negatives included. Pertinent negatives are of particular importance, since ordering providers cannot assume that omission of a finding from a report constitutes absence of the finding, especially with findings that can predispose patients to surgical complications (20). Radiologists can use this framework to supplement their established protocols for reviewing temporal bone CTs, using preoperative clinical correlates to specifically highlight features in their report that may be relevant for surgeons. Surgeons may benefit from reviewing preoperative imaging more systematically using this tool so as not to miss key anatomical variants that may impact surgical approach or risk.

**Table 1:** A systematic approach to reviewing computed tomography of the temporal bone using the mnemonic “**M**aking **M**iracles **I**nvolves **F**ocused **V**ision.”.

Region	Findings	Clinical/Surgical Implications
*Mastoid*	• Is the mastoid well-pneumatized? Are air cells present? • Is the mastoid sclerotic or contracted? • Is there opacification within the mastoid? Is there bony erosion of coalescence of air cells? • Is the **tegmen** height normal or low? Is the tegmen mastoideum intact without defects, fractures, or irregularities?	• Poor pneumatization/sclerosis can blunt anatomical landmarks and lead to a contracted surgical field • Fluid may imply mastoid effusion/opacification, or if coalescent, mastoiditis • Low lying tegmen can limit exposure to the antrum and increase risk of dural injury
*Middle Ear*	• Is the **ossicular chain** intact? Are there any malformations or erosions? Are there findings suggestive of ossicular fixation (e.g., tympanosclerosis, ossicular thickening, ankylosis, or otosclerotic foci)? • Is the middle ear well aerated? Is the **tympanic membrane** retracted or thickened? • Is there fluid or soft tissue opacification within the middle ear? Is there erosion adjacent to soft tissue? • Is there evidence of irregular or hypodense bone or otospongiosis, especially at the **fissula ante fenestram**? What is the Veillon or Symons and Fanning classification stage?	• Conductive hearing loss and need for ossicular chain reconstruction or stapedectomy • Poor aeration and Eustachian tube dysfunction can portend poor outcome with tympanoplasty • Confirming diagnosis and severity of suspected otosclerosis • Soft tissue with erosion (classically at the scutum) raises suspicion for cholesteatoma
*Inner Ear*	• Examine the **cochlea**. Is there malformation, sclerosis, or ossification? If a malformation is identified, what is the specific malformation (e.g., Sennaroglu classification)? • Is the **vestibular aqueduct** enlarged? (Cincinnati criteria: vestibular aqueduct midpoint >0.9 mm and operculum >1.9 mm; Valvassori and Clemis criteria: midpoint greater than or equal to 0.5 mm). • Are the **oval and round windows** patent or hypoplastic? • Is the **cochlear aperture** of normal size, or is there evidence of cochlear nerve canal hypoplasia/atresia? Is the IAC expanded? • Survey the **vestibule and semicircular canals**. Are there bony dehiscences or malformations?	• Clarify etiology of sensorineural or congenital hearing loss • Establish cochlear implant candidacy. Specific IEMs may preclude CI placement. Other IEMs impact CI electrode choice and technical approach • EVA may increase risk of CSF leak or intraoperative gusher • Cochlear fibrosis or labyrinthitis ossificans can make electrode insertion difficult or impossible, necessitating modifications such as subtotal petrosectomy, split electrode arrays, retrograde insertion, scala vestibuli insertion, or middle/apical cochleostomy • Cochlear aperture stenosis may impact CI outcomes but is not an absolute contraindication to CI • Enlarged IAC may indicate neoplasm, most commonly vestibular schwannoma • Semicircular canal dehiscence is seen in SSCD syndrome, however not all dehiscences lead to symptoms
*Facial Nerve*	• Examine the **course of the facial nerve**. Does it follow a normal course or aberrant course? Is it anteriorly or laterally displaced? Does it overhang the oval window? • Is the facial nerve covered in bone or are there regions of bony dehiscence? • Examine the **facial recess**. Is the facial recess width (distance between facial nerve and tympanic annulus at the level of the basal turn of the cochlea and round window) >2 mm?	• Aberrant facial nerve course increases risk of intraoperative injury • Facial nerve course and any deviation from typical must be studied prior to any otologic surgery • Modified surgical approaches may be needed with aberrant facial nerve course, such as retrofacial, trans-attic combined with transcanal, or facial recess combined with transcanal approaches
*Vascular*	• Is the **sigmoid sinus** dominant, enlarged, lateral, or anterior? • What is the location of the **jugular bulb**? Is it high-riding? Is it enlarged? Any dehiscence or jugular diverticula? • Does the **carotid artery** follow the expected course? • Any evidence of a **persistent stapedial artery** (a linear soft tissue density crossing over the cochlear promontory and through the stapes crura)?	• High-riding jugular bulb or anterior sigmoid sinus constrains surgical access, rarely necessitating canal wall down or other approaches • Lateral sigmoid sinus at higher risk of injury • Difficult to control and rarely catastrophic bleeding can occur • A persistent stapedial artery is an absolute contraindication to stapes surgery and most ossicular chain reconstruction

## Mastoid

The mastoid portion of the temporal bone is evaluated first. A healthy mastoid is characterized by well-pneumatized air cells that allow clear visualization of the surrounding bony structures on CT. Normal mastoid pneumatization appears as a network of air-filled spaces within the bone, with an intact cortical boundary (21). The mastoid is evaluated for aeration/pneumatization, sclerosis, and the presence of fluid. The integrity and height of the tegmen mastoideum, which forms the middle cranial fossa floor, is also evaluated. A normal tegmen is smooth, without evidence of defects or encephalocele (22).

**Figure 1:**
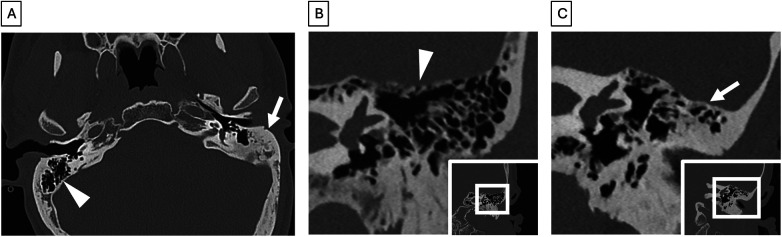
Mastoid anatomy (all images represent different patients). Axial CT shows highly pneumatized mastoid air cells on the right (arrowhead) and sclerotic air cells on the left (arrow)​. **(A)** Photon-counting coronal CT shows normal tegmen mastoideum (arrowhead) height. **(B)** Photon-counting coronal CT shows reduced tegmen mastoideum (arrow) height.

Opacification in the mastoid can occur with serous effusion, trauma, and otitis media or mastoiditis. An important distinction for clinicians is the presence not only of mastoid opacification but also bony erosion and mastoid air cell coalescence, which defines a true, infectious mastoiditis rather than a sterile effusion or chronic fibrosis (23). Most otolaryngologists would argue that mastoid opacification without coalescence or bony erosion should be termed “mastoid opacification” on the imaging report rather than “mastoiditis.” Chronic or recurrent opacification from infection or Eustachian tube dysfunction and bony erosion over time can lead to reduced or absent pneumatization, which leads to a sclerotic and contracted mastoid (Figure 1A) (24, 25). This is important to recognize before and during mastoidectomy, as anatomy within a sclerotic mastoid with reduced pneumatization is more difficult to read, which increases risk to nearby anatomical structures.

A sclerotic, contracted mastoid can also lead to poor access to ear structures such as the semicircular canals, epitympanum, and cochlea (26). Contraction can cause sagging of the tegmen, effectively lowering the floor of the middle fossa and decreasing access superiorly, increasing the risk of tegmen injury which can lead to dural injury, CSF leak, temporal lobe damage, or encephalocele (Figure 1C) (14, 26, 27). It can also lead to a more anteriorly displaced sigmoid sinus. In severe cases, this restricts access to the cochlea through a facial recess approach, necessitating a canal wall down approach to place a cochlear implant without compromising the tegmen, sigmoid sinus, and facial nerve. The tegmen mastoideum can also be dehiscent and contain large defects with encephalocele or meningoencephalocele. It is important to be aware of these anatomical variants preoperatively for surgical planning as well as patient counseling.

## Middle ear

**Figure 2:**
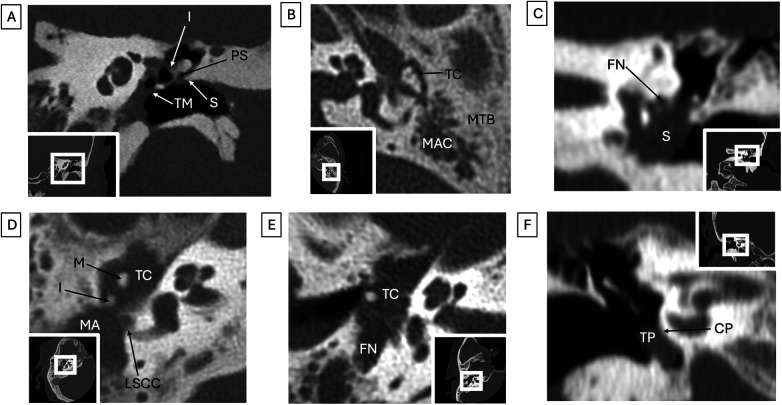
Middle ear pathology (all images represent different patients). **(A)** Cholesteatoma. Photon-counting coronal CT shows soft tissue opacification within the epitympanum, including involving Prussak's space (PS), and with erosion of the medial aspect of the incus (I) and retraction of the tympanic membrane (TM). No erosion of the scutum (S). **(B)** Chronic otitis media. Axial CT shows complete opacification of the left tympanic cavity (TC) and mastoid air cells (MAC) with marked sclerosis of the mastoid part of the temporal bone (MTB). **(C)** Facial nerve schwannoma. Coronal CT shows an intratympanic soft tissue mass (S) which contacts the tympanic facial nerve canal (FN). **(D)** Metastasis. Axial CT shows a soft tissue mass filling the tympanic cavity (TC) and mastoid antrum (MA) with erosion and dissociation of the malleus (M) and incus (I) as well as dehiscence of the lateral semicircular canal (LSCC) in a patient with metastatic non-small cell lung cancer. **(E)** Chronic idiopathic polyneuropathy. Axial CT shows marked expansion of the mastoid facial nerve canal (FN) with remodeling, expansion, and soft tissue opacification of the tympanic cavity (TC) due to enlargement of the facial nerve and chorda tympani. **(F)** Tympanic paraganglioma. Coronal CT shows an ovoid soft tissue density (TP) within the tympanic cavity abutting the cochlear promontory (CP) near the expected location of the tympanic branch of cranial nerve IX.

The middle ear space includes the region from the tympanic membrane laterally to the bony labyrinth medially, as well as all the air-filled spaces around these structures. Middle ear aeration, opacification, tegmen tympani integrity, and the ossicular chain should be evaluated in the imaging report. Many pathologies can affect the middle ear including trauma, Eustachian tube dysfunction, otitis media, cholesteatoma, otosclerosis, and middle ear neoplasms (Figure 2).

The ossicular chain, comprised of the malleus, incus, and stapes, should appear intact, well aligned, and without evidence of malformations or erosion (28). Malformations may arise at any stage of embryological development as the auditory ossicles are formed from the first and second pharyngeal arches, leading to isolated congenital middle ear anomalies (CMEAs) (29). Middle ear anomalies are also associated with congenital syndromes, including Stickler, Treacher Collins, Crouzon, CHARGE, and branchio-oto-renal syndrome (30).

Poor middle ear ventilation from Eustachian tube dysfunction can lead to inflammation and recurrent or chronic otitis media as well as cholesteatoma (Figure 2A). This can lead to ongoing inflammation with opacification and bony erosion (31, 32). Ossicle erosion can cause conductive hearing loss, with eventual ossicular discontinuity. The presence of soft tissue opacification in the middle ear can be difficult to interpret, as CT is unable to distinguish cholesteatoma from granulation tissue, scar, or other soft tissue. However, the presence of bony erosion, especially blunting at the scutum, a sharp bony spur that is formed by the superior wall of the external auditory canal and the lateral wall of the tympanic cavity that forms the lateral margin of Prussak's space, increases the diagnostic suspicion for cholesteatoma (33). When imaging is suggestive of cholesteatoma, this is an indication for surgery.

**Figure 3:**
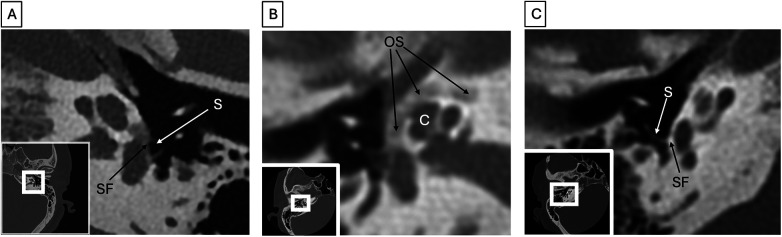
Otosclerosis (all images represent different patients). **(A)** Fenestral otosclerosis. Photon-counting axial CT shows lucency at the fissula ante fenestram (FAF), which extends to the stapes footplate (SF) along the anterior crus of the stapes (S). **(B)** Retrofenestral otosclerosis. Axial CT shows circumferential lucency (OS) about the cochlea **(C)**, producing a “double ring” sign. **(C)** Ossicular chain variation. Axial CT of a patient with otosclerosis shows a monocrural stapes (S) which contacts a markedly thickened stapes footplate (SF).

Otosclerosis is a common indication for ear surgery, namely stapedotomy or stapedectomy, or cochlear implantation in more advanced cases. This autosomal dominant otodystrophy of the otic capsule is characterized by replacement of normal ivory-like endochondral bone by spongy vascular bone, termed otospongiosis (34). Over time this bone may re-calcify and become more solid and sclerotic. Otosclerosis is categorized into two types, fenestral and retrofenestral, depending on the pattern of otic capsule bony involvement. The more common fenestral type is characterized by involvement of the lateral wall of the bony labyrinth, classically at the fissula ante fenestram, a cleft of fibrocartilaginous tissue between the inner and middle ear, just anterior to the oval window (Figure 3A) (34). The cochlear promontory, round window niche, and stapes footplate can be involved, eventually leading to stapes fixation, which in turn can impact the ossicular chain with incus subluxation or malleo-incudal dislocation. Bilateral involvement is common. In retrofenestral otosclerosis, typically later in the continuum of the otoslcerotic process, there is involvement of the cochlea with associated sensorineural hearing loss (Figure 3B).

**Figure 4:**
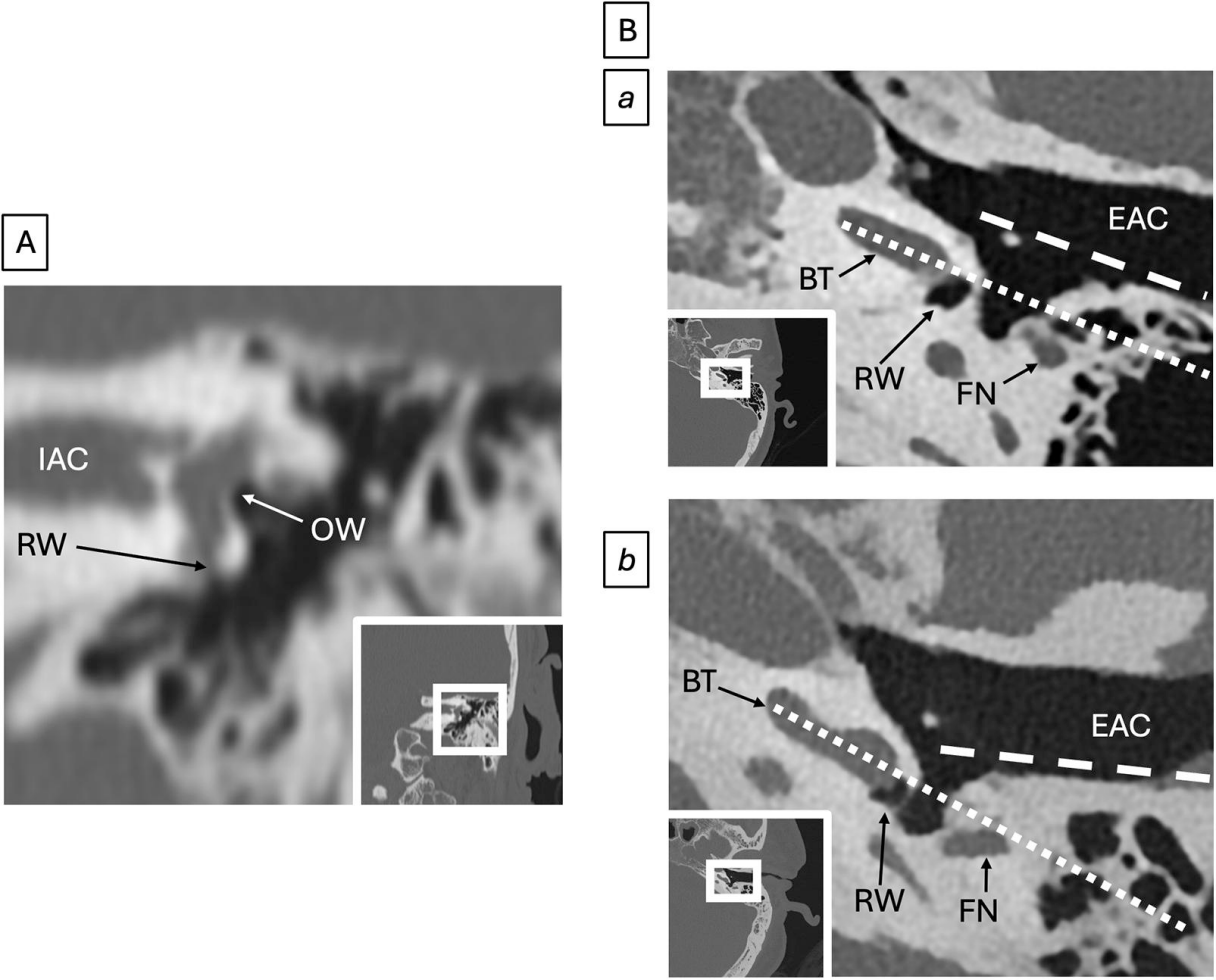
Round window anatomy (all images represent different patients). **(A)** Round window stenosis. Coronal CT in this patient with otosclerosis shows bony overgrowth causing severe stenosis of the round window (RW). Oval window (OW) and internal auditory canal (IAC) also shown for reference. **(B)** Photon-counting axial CT in patient (a) and patient (b) show a more favorable surgical approach in (a) compared to (b), as the long axis (dotted line) of the basal turn of the cochlea (BT) in patient (a) is parallel to the long axis (dashed line) of the posterior wall of the external auditory canal (EAC). Round window (RW) and mastoid facial nerve canal (FN) are provided for reference.

High-resolution CT may demonstrate patchy otospongiotic bone in the cochlea or a pericochlear hypodense double ring (also called the fourth ring of Valvassori) (35). Imaging is often not pursued in patients with characteristic clinical and audiometric findings of otosclerosis (Carhart notch, absent stapedial reflexes, air bone gap >30–40 dB). However, in equivocal or complicated cases imaging can be of great value. Radiologists should assess the size and location of otospongiotic plaques, the status of the oval and round windows, and the facial nerve canal. Otosclerosis can lead to ossicular chain variation including a monocrural stapes (Figure 3C). Round window obliteration and otospongiosis are important to note as this can reduce the efficacy of stapedectomy (Figure 4A) (8). Additionally, it may be helpful to include a radiological staging classification system for otosclerosis such as the Veillon or the Symons-Fanning CT Classification System (36, 37). The Veillon system classifies otosclerosis by Type Ia, Ib, II, III, IVa, and IVb, based on the degree of footplate, fenestral, and otic capsule hypodensity (36). Both staging systems have been correlated with pre- and postoperative pure tone audiometric data (38).

Various middle ear masses can also be seen on temporal bone CT (Figure 2). These include tympanic paraganglioma, a highly vascular middle ear paraganglioma that can present clinically with pulsatile tinnitus and conductive hearing loss (Figure 2F) (39). On otoscopic examination, a reddish hue may be observed behind the tympanic membrane, with blanching of the mass on pneumatic otoscopy termed Brown's sign. Facial schwannoma, metastatic masses, and changes from systemic chronic diseases (such as chronic idiopathic polyneuropathy, Paget's disease, osteogenesis imperfecta, and others) can also be observed in the middle ear (Figures 2C–E).

## Inner ear & internal auditory canal (IAC)

**Figure 5:**
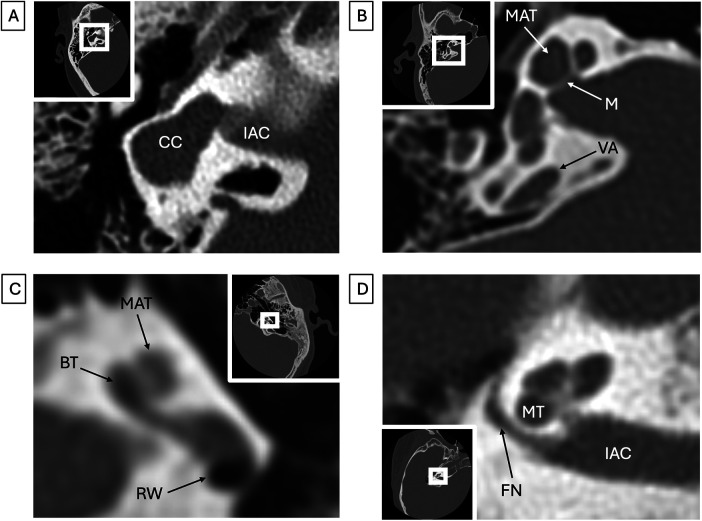
Inner ear congenital abnormalities (all images represent different patients). **(A)** Common cavity malformation. Axial CT shows a common cavity (CC) comprising both the vestibule and cochlea, which communicates with a dysplastic enlarged internal auditory canal (IAC). **(B)** Incomplete partition (IP). Axial CT in a patient with IP type II shows coalescence of the middle and apical turns (MAT), a deficient modiolus (M), and an enlarged vestibular aqueduct (VA). Round window (RW) is shown for reference. **(C)** Cochlear hypoplasia. Axial CT in a patient with branchio-oto-renal syndrome shows a dysplastic “unwound” basal turn (BT) and hypoplasia of the apical and middle turns (MAT). **(D)** Aberrant facial nerve course. Axial CT shows aberrant course of the labyrinthine segment of the facial nerve (FN) extending posterior to the middle turn of the cochlea (MT) in a patient with CHARGE syndrome. Internal auditory canal (IAC) is shown for reference.

Inner ear structures include the cochlea, vestibule, semicircular canals, and the internal auditory canal (IAC). These microscopic structures must be carefully assessed for variations in anatomy, malformations, and dehiscence. Detailed assessment of the cochlea is essential in cases of hearing loss and prior to cochlear implantation (CI). The cochlea is examined for severe malformations, sclerosis or ossification, and cochlear nerve deficiency. Inner ear malformations (IEMs) exist on a continuum, classified by Sennaroglu, with the most severe cases showing complete labyrinthine aplasia (CLA), also called Michel deformity, or isolated cochlear aplasia (Figure 5) (40). CLA is characterized by complete absence of labyrinthine structures, an atretic IAC, and an aberrant course of the facial nerve with normal or dysplastic ossicles. CLA is an absolute contraindication to CI placement (10). Common cavity malformation occurs when the cochlea and vestibule form a single chamber (Figure 5A). CI placement is possible in common cavities, but it can be challenging to place electrodes close to neural elements and outcomes are more variable (20). Cochlear hypoplasia (CH) occurs across a spectrum, from a bud-like cochlea (type I), cystic hypoplastic cochlea (type II), cochlea with less than 2 turns (type III), and cochlea with a normal basal turn but hypoplastic middle and apical turns (type IV) (Figure 5C) (41). In cases of incomplete partition (IP) of the cochlea, the cochlea has normal dimensions in contrast to CH where it is smaller. In IP type I, there is no modiolus or interscalar septum. IP type II may be accompanied by an enlarged vestibular aqueduct, which when found together is called a Mondini malformation (Figure 5B). IPs and EVAs are important to identify as they not only impact electrode choice and audiometric outcomes but can also increase the risk for cerebrospinal fluid leak or intraoperative “gusher.” A defective stapes footplate increases the risk of a perilymphatic fistula and meningitis before surgery in a few IEMs, including common cavity malformation, CH type II, IP type I, and IP type III (10). EVA can also occur in isolation, with isolated EVA being the most common congenital anomaly associated with sensorineural hearing loss (10). Several formal criteria are frequently used to define an EVA, including the Cincinnati criteria (vestibular aqueduct midpoint >0.9 mm and operculum >1.9 mm) and the Valvassori and Clemis criteria (midpoint greater than or equal to 0.5 mm). The caliber of the posterior semicircular canal is usually similar to that of a non-enlarged vestibular aqueduct and can be used as a quick check. While a complete review of inner ear malformations is out of the scope of this review, standardized reporting of IEMs using a clear classification scheme such as that set forth by Sennaroglu is highly recommended for consistency and clarity.

**Figure 6:**
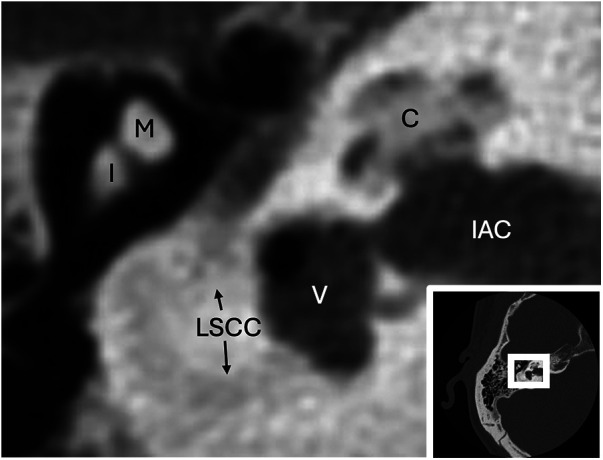
Labyrinthitis ossificans. Axial CT shows extensive ossification of the cochlea (C) and lateral semicircular canal (LSCC) due to severe labyrinthitis ossificans. Vestibule, malleus head (M), incus body (I), and internal auditory canal (IAC) are provided for reference.

Oval and round window patency and hypoplasia should be recognized, as poor development of these structures can increase intraoperative difficulty and may preclude round window electrode insertion (Figure 4) (42). Chronic otitis media, temporal bone trauma, Cogan's syndrome, and otosclerosis may cause cochlear fibrosis or labyrinthitis ossificans, which can make electrode insertion difficult or impossible, necessitating technique modifications such as subtotal petrosectomy, split electrode arrays, retrograde insertion, scala vestibuli insertion, or middle/apical cochleostomy (Figure 6) (43).

The cochlear aperture and the IAC should be assessed prior to cochlear implantation and to rule out nerve abnormalities and retrocochlear pathology including IAC masses. Cochlear nerve canal hypoplasia/atresia is defined by a canal diameter at the modiolus of 1.5 mm or less and is often associated with cochlear nerve hypoplasia or aplasia. Cochlear implantation is controversial in patients with cochlear nerve aplasia. However, cochlear aperture stenosis is not an absolute contraindication to CI, and some patients can still have good speech comprehension outcomes after implantation even with cochlear aperture stenosis. MRI is the gold standard modality for diagnosing cochlear nerve abnormalities, but CT imaging can often provide clues. The IAC is a narrow, conical bony passageway extending from the porus acusticus to the fundus that contains critical neurologic structures including the facial nerve, cochlear nerve, and the superior and inferior vestibular nerves. The most common tumor of the temporal bone, vestibular schwannomas, arise from the IAC.

**Figure 7:**
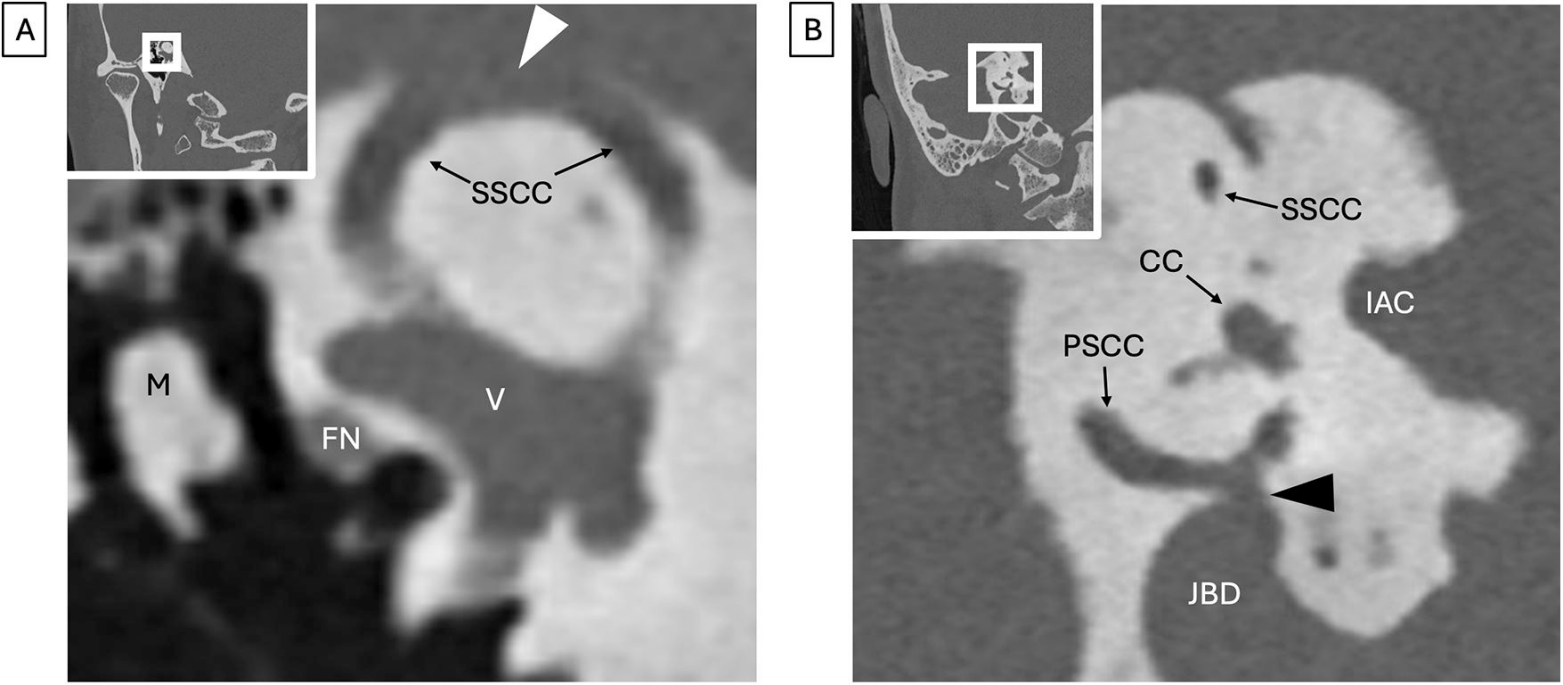
Third window defects (both images represent different patients). **(A)** Photon-counting Pöschl view CT shows dehiscence (arrowhead) along the roof of the superior semicircular canal (SSCC). Vestibule (V), head of the malleus (M), and tympanic facial nerve canal (FN) are provided for reference. **(B)** Photon-counting Stenvers view CT shows dehiscence (arrowhead) of the posterior semicircular canal (PSCC) adjacent to a jugular bulb diverticulum (JBD). Superior semicircular canal (SSCC), common crus (CC), and internal auditory canal provided for reference.

Survey of the vestibule and semicircular canals in important, especially in cases of suspected semicircular canal dehiscence. If a third window defect is suspected, reformatted planes aligned to the superior semicircular canal (Stenvers and Pöschl projections) can provide optimal visualization of the superior semicircular canal, enabling early detection of bone thinning or dehiscence that may be missed and enhance preoperative planning by offering detailed anatomical views (44) (Figure 7).

## Facial nerve

**Figure 8:**
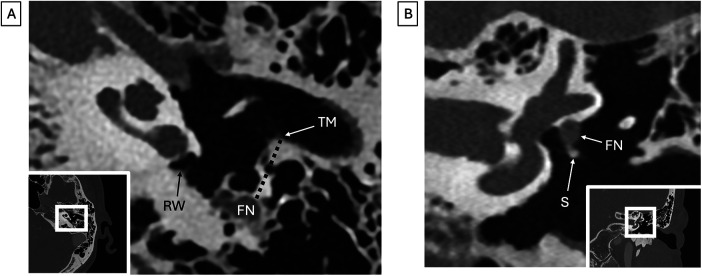
Facial nerve anatomy (both images are from the same patient). **(A)** Photon counting axial CT shows a normal facial recess width (dotted line), measured from the mastoid segment of the facial nerve (FN) to the annulus of the tympanic membrane (TM) at the level of the round window (RW). **(B)** Overhanging facial nerve. Photon counting coronal CT image shows dehiscence of the facial nerve canal with extension of the tympanic segment of the facial nerve (FN) into the tympanic cavity, with contact of the stapes (S).

The intratemporal facial nerve typically follows a well-defined path through the temporal bone from the porus acusticus to the stylomastoid foramen. The course of the nerve, and any variations from the expected course or any bony dehiscence, are of paramount importance to recognize prior to any otologic surgery. Tympanic segment dehiscence places the facial nerve at elevated risk of injury with middle ear surgery and must be recognized. Abnormal anterior or lateral displacement of the facial nerve increases the risk of nerve injury and can lead to technical challenges. The presence of IEMs often occurs in tandem with abnormal position of the facial nerve (Figure 4D). Many variants have been described. An overhanging facial nerve deviates medially toward the oval window, which increases the risk of nerve injury during mastoidectomy, stapedectomy, or cochlear implantation (Figure 8B) (45). Modified surgical approaches may be needed with aberrant facial nerve course, such as retrofacial, trans-attic combined with transcanal, or facial recess combined with transcanal approaches (46).

In CI surgery, the cochlea is most often accessed via the facial recess. The facial nerve and the chorda tympani nerve form the medial and lateral walls of the facial recess. A facial recess width (distance between facial nerve and tympanic annulus at the level of the basal turn of the cochlea and round window) of greater than 2 mm and the presence of herald air cells in this region are associated with easy accessibility with posterior tympanotomy (Figures 4B, 8A) (10). Identifying these variants is crucial, as damage to the facial nerve can result in permanent facial paralysis, significantly impacting quality of life.

## Vascular structures

**Figure 9:**
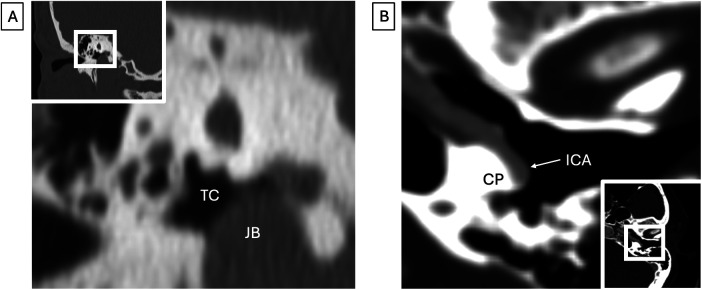
Carotid and jugular anatomy (both images represent different patients). **(A)** Jugular bulb variation. Coronal CT shows a dehiscent high-riding jugular bulb (JB) protruding into the tympanic cavity (TC). **(B)** Carotid artery variation. Axial CTA shows an aberrant left internal carotid artery (ICA) coursing across the cochlear promontory (CP).

Several large vascular structures pass through the temporal bone, and inadvertent damage during surgery can lead to significant morbidity or fatal hemorrhage. Common venous variations include an anterior and/or lateral sigmoid sinus and a high-riding jugular bulb, both of which constrict the operative field during mastoidectomy and can interfere with surgical access (Figure 9A). In such cases, the jugular bulb can be skeletonized, mobilized, or decompressed if absolutely necessary to access adjacent structures. However, these techniques can induce significant jugular bleeding, requiring intraoperative control with various hemostasis methods including compression and the use of hemostatic agents. The position of these vascular structures should be commented on in every temporal bone CT report. Classification schema for jugular bulb position and sigmoid sinus position have been described (47, 48). The jugular bulb can also be enlarged, dehiscent, or contain jugular diverticula.

A persistent stapedial artery results from failure of regression of the embryonic stapedial artery and is associated with a linear soft tissue density crossing over the cochlear promontory and through the stapes crura, with an aplastic or hypoplastic foramen spinosum. This rare variant is a contraindication to stapes surgery and is associated with an aberrant carotid artery. An aberrant carotid artery can result from absence of the cervical ICA, leading to persistent inferior tympanic and caroticotympanic arteries over the cochlear promontory (Figure 9B) (49). Identifying these vascular anomalies on CT allows the surgeon to assess surgical candidacy and risk and to proactively plan for modifications in technique and approach.

## Limitations

This review has several limitations. This is a narrative review based on expert consensus at a single institution and therefore has not been rigorously validated to the extent that would be possible through a large committee of experts. This mnemonic provides a general educational framework with case images and references but otherwise does not introduce original data or analyses. The mnemonic itself, while useful as a framework for reviewing imaging, is slightly long and less useful to non-native English speakers. Future validation with interobserver agreement and report completeness among a larger panel of experts to establish consensus guidelines is needed.

## Conclusion

A detailed preoperative CT of the temporal bone is an essential part of planning for otologic surgery. Understanding the normal anatomy, as well as common and rare anatomical variants, can significantly improve surgical outcomes and reduce complications. The systematic approach outlined in this narrative review, using the mnemonic “**M**aking **M**iracles **I**nvolves **F**ocused **V**ision,” provides a comprehensive framework for evaluating temporal bone CTs. By recognizing critical landmarks and anatomic variations, radiologists can inform clinicians and surgeons can tailor their approach to each patient's unique anatomy, ensuring safer and more effective surgical intervention.

## References

[1] Flint PW Haughey BH Lund VJ Niparko JK Robbins KT Thomas JR Cummings Otolaryngology: Head and Neck Surgery - ClinicalKey. Available online at: https://www.clinicalkey.com/#!/browse/book/3-s2.0-C20161050836 (Accessed May 13, 2025).

[2] Anson BJ Donaldson JA Anson BJ Donaldson JA. Surgical Anatomy of the Temporal Bone. 3rd ed. Philadelphia, PA: WB Saunders Company (1981).

[3] Aslan A Mutlu C Celik O Govsa F. Ozgur T. Egrilmez M. Surgical implications of anatomical landmarks on the lateral surface of the mastoid bone. Surg Radiol Anat. (2004) 26:263–7. 10.1007/s00276-004-0235-1 15205917

[4] Moroco AE Saadi RA Baker AR Zhu J Isildak H. Usage patterns of CT and MRI in the evaluation of otologic disease. Otol Neurotol. (2021) 42(6):e698. 10.1097/MAO.0000000000003095 33606467

[5] Corrales CE Fischbein N Jackler RK. Imaging innovations in temporal bone disorders. Otolaryngol Clin North Am. (2015) 48(2):263–80. 10.1016/j.otc.2014.12.002 25769351

[6] Casselman JW. Diagnostic imaging in clinical neuro-otology. Curr Opin Neurol. (2002) 15(1):23. Available online at: https://journals.lww.com/co-neurology/fulltext/2002/02000/diagnostic_imaging_in_clinical_neuro_otology.5.aspx (Accessed January 7, 2025) 10.1097/00019052-200202000-00005 11796947

[7] Chen JY Mafee MF. Computed tomography imaging technique and normal computed tomography anatomy of the temporal bone. Oper Tech Otolaryngol-Head Neck Surg. (2014) 25(1):3–12. 10.1016/j.otot.2013.11.002

[8] Purohit B Hermans R Op de beeck K. Imaging in otosclerosis: a pictorial review. Insights Imaging. (2014) 5(2):245–52. 10.1007/s13244-014-0313-9 24510845 PMC3999364

[9] Davidson HC. Imaging evaluation of sensorineural hearing loss. Semin Ultrasound CT MRI. (2001) 22(3):229–49. 10.1016/S0887-2171(01)90009-5 11451098

[10] Hiremath SB Biswas A Mndebele G Schramm D Ertl-Wagner BB Blaser SI Cochlear implantation: systematic approach to preoperative radiologic evaluation. RadioGraphics. (2023) 43(4):e220102. 10.1148/rg.220102 36893052

[11] Branstetter BF Harrigal Chivonne Escott Edward J. Hirsch Barry E. Superior semicircular canal dehiscence: oblique reformatted CT images for diagnosis. Radiology. (2006) 238(3):938–42. 10.1148/radiol.2382042098 16424241

[12] Benson JC Rajendran K Lane JI Diehn FE Weber NM Thorne JE A new frontier in temporal bone imaging: photon-counting detector CT demonstrates superior visualization of critical anatomic structures at reduced radiation dose. AJNR Am J Neuroradiol. (2022) 43(4):579–84. 10.3174/ajnr.A7452 35332019 PMC8993187

[13] Hermans R Boomgaert L Cockmartin L Binst J De Stefanis R Bosmans H. Photon-counting CT allows better visualization of temporal bone structures in comparison with current generation multi-detector CT. Insights Imaging. (2023) 14(1):112. 10.1186/s13244-023-01467-w 37395919 PMC10317909

[14] Kennedy KL Lin JW. Mastoidectomy. In: StatPearls. Treasure Island (FL): StatPearls Publishing (2025). Available online at: http://www.ncbi.nlm.nih.gov/books/NBK559153/ (Accessed January 7, 2025).

[15] Deep NL Dowling EM Jethanamest D Carlson ML. Cochlear implantation: an overview. J Neurol Surg Part B Skull Base. (2019) 80(2):169–77. 10.1055/s-0038-1669411 PMC6438790 30931225

[16] Toscano ML Shermetaro C. Stapedectomy. In: StatPearls. Treasure Island (FL): StatPearls Publishing. Available online at: http://www.ncbi.nlm.nih.gov/books/NBK562205/2025 (Accessed January 7, 2025).

[17] Indorewala S Adedeji TO Indorewala A Nemade G. Tympanoplasty outcomes: a review of 789 cases. Iran J Otorhinolaryngol. (2015) 27(79):101–8.PMCID: PMC4409954, PMID: 25938081 25938081 PMC4409954

[18] Sheikh MM De Jesus O. Vestibular schwannoma. In: StatPearls. Treasure Island (FL): StatPearls Publishing (2025). Available online at: http://www.ncbi.nlm.nih.gov/books/NBK562312/ (Accessed January 13, 2025). 32965983

[19] O’Brien WT Hamelin S Weitzel EK. The preoperative Sinus CT: avoiding a “CLOSE” call with surgical complications. Radiology. (2016) 281(1):10–21. 10.1148/radiol.2016152230 27643765

[20] Widmann G Dejaco D Luger A Schmutzhard J. Pre- and post-operative imaging of cochlear implants: a pictorial review. Insights Imaging. (2020) 11:93. 10.1186/s13244-020-00902-6 32803542 PMC7429612

[21] Deutschmann MW Yeung J Bosch M Lysack John T. Kingstone Michael Kilty Shaun J. Radiologic reporting for paranasal sinus computed tomography: a multi-institutional review of content and consistency. Laryngoscope. (2013) 123(5):1100–5. 10.1002/lary.23906 23619621

[22] Khosravi M Jabbari Moghaddam Y Esmaeili M Keshtkar A Jalili J Tayefi Nasrabadi H. Classification of mastoid air cells by CT scan images using deep learning method. J Big Data. (2022) 9(1):62. 10.1186/s40537-022-00596-1

[23] Makki FM Amoodi HA van Wijhe RG Bance M. Anatomic analysis of the mastoid tegmen: slopes and tegmen shape variances. Otol Neurotol. (2011) 32(4):581. 10.1097/MAO.0b013e31820e75f7 21765385

[24] Ilea A Butnaru A Sfrângeu SA Hedeşiu M Dudescu CM Berce P Role of mastoid pneumatization in temporal bone fractures. AJNR Am J Neuroradiol. (2014) 35(7):1398–404. 10.3174/ajnr.A3887 24610903 PMC7966584

[25] Minks DP Porte M Jenkins N. Acute mastoiditis—the role of radiology. Clin Radiol. (2013) 68(4):397–405. 10.1016/j.crad.2012.07.019 22980753

[26] Rodrigues H Ramos R Fagundes L Galego O Navega D Coelho JD Mastoid, middle ear and inner ear analysis in CT scan—a possible contribution for the identification of remains. Med Sci Law. (2020) 60(2):102–11. 10.1177/0025802419893424 32050849

[27] Woolley AL Oser AB Lusk RP Bahadori RS. Preoperative temporal bone computed tomography scan and its use in evaluating the pediatric cochlear implant candidate. Laryngoscope. (1997) 107(8):1100–6. 10.1097/00005537-199708000-00017 9261015

[28] Husain M Khanduri S Faiz SM Abbas SZ Yadav P Khan AU Role of HRCT temporal bone in Pre-operative assessment of tegmen height in chronic Otitis Media patients. J Clin Imaging Sci. (2020) 10:79. 10.25259/JCIS_131_2020 33365201 PMC7749932

[29] Lemmerling MM De Foer B VandeVyver V Vercruysse JP Verstraete KL. Imaging of the opacified middle ear. Eur J Radiol. (2008) 66(3):363–71. 10.1016/j.ejrad.2008.01.020 18339504

[30] Park K Choung YH. Isolated congenital ossicular anomalies. Acta Otolaryngol. (2009) 129(4):419–22. 10.1080/00016480802587846 19116789

[31] Benson JC Diehn F Passe T Guerin J Silvera VM Carlson ML The forgotten second window: a pictorial review of round window pathologies. AJNR Am J Neuroradiol. (2020) 41(2):192–9. 10.3174/ajnr.A6356 31831467 PMC7015198

[32] Gettelfinger JD Dahl JP. Syndromic hearing loss: a brief review of common presentations and genetics. J Pediatr Genet. (2018) 7(1):1–8. 10.1055/s-0037-1617454 29441214 PMC5809162

[33] Juliano AF Ginat DT Moonis G. Imaging review of the temporal bone: part I. Anatomy and inflammatory and neoplastic processes. Radiology. (2013) 269(1):17–33. 10.1148/radiol.13120733 24062560

[34] Corrales CE Blevins NH. Imaging for evaluation of cholesteatoma: current concepts and future directions. Curr Opin Otolaryngol Head Neck Surg. (2013) 21(5):461–7. 10.1097/MOO.0b013e328364b473 23880648

[35] Valvassori GE. Imaging of otosclerosis. Otolaryngol Clin North Am. (1993) 26:359–71. 10.1016/S0030-6665(20)30815-X 8341568

[36] Veillon F Riehm S Emachescu B Haba D Roedlich MN Greget M Imaging of the windows of the temporal bone. Semin Ultrasound CT MRI. (2001) 22(3):271–80. 10.1016/S0887-2171(01)90011-3 11451100

[37] Marshall AH Fanning N Symons S Shipp D Chen JM Nedzelski JM. Cochlear implantation in cochlear otosclerosis. Laryngoscope. (2005) 115(10):1728–33. 16222185 10.1097/01.mlg.0000171052.34196.ef

[38] Pinto JV Almeida AI Andrade A Vales F Moura CP Marques P. Comparison between the veillon and the symons–fanning CT classification systems for otosclerosis. Otol Neurotol. (2024) 45(9):e618. 10.1097/MAO.0000000000004311 39264917

[39] Appannan VR Daud M K M. Glomus tympanicum. Malays Fam Physician. (2018) 13(1):45–8.PMCID: PMC5962235, PMID: 29796211 29796211 PMC5962235

[40] Sennaroglu L Saatci I. A new classification for cochleovestibular malformations. Laryngoscope. (2002) 112(12):2230–41. 10.1097/00005537-200212000-00019 12461346

[41] Sennaroglu L. Cochlear implantation in inner ear malformations—a review article. Cochlear Implants Int. (2010) 11:4–41. 10.1002/cii.416 19358145

[42] Birman CS Brew JA Gibson WPR Elliott EJ. CHARGE Syndrome and cochlear implantation: difficulties and outcomes in the paediatric population. Int J Pediatr Otorhinolaryngol. (2015) 79:487–92. 10.1016/j.ijporl.2015.01.004 25649713

[43] Dejaco D Prejban D Fischer N Freysinger Wolfgang Stephan Kurt Seebacher Josef Successful cochlear implantation of a split electrode array in a patient with far-advanced otosclerosis assisted by electromagnetic navigation: a case report. Otol Neurotol. (2018) 39:e532–7. 10.1097/MAO.0000000000001845 29995006

[44] Ceylan N Bayraktaroglu S Alper H Savaş R Bilgen C Kirazli T CT Imaging of superior semicircular canal dehiscence: added value of reformatted images. Acta Otolaryngol (Stockh). (2010) 130(9):996–1001. 10.3109/00016481003602108 20205621

[45] Gupta S Mends F Hagiwara M Fatterpekar G Roehm PC. Imaging the facial nerve: a contemporary review. Radiol Res Pract. (2013) 2013:248039. 10.1155/2013/248039 23766904 PMC3676972

[46] Telmesani LM Alrammah MK. Telmesani radiological classification of the location of the vertical segment of the facial nerve: impact on surgical approach in cochlear implant surgery. Otol Neurotol. (2017) 38:e335–8. 10.1097/MAO.0000000000001547 28820756

[47] Manjila S Bazil T Kay M Udayasankar UK Semaan M. Jugular bulb and skull base pathologies: proposal for a novel classification system for jugular bulb positions and microsurgical implications. Neurosurg Focus. (2018) 45(1):E5. 10.3171/2018.5.FOCUS18106 29961385

[48] Mandour M Tomoum M El Zayat S Hamad H Amer M. Surgeon oriented preoperative radiologic evaluation in cochlear implantation: our experience with a proposed checklist. Int Arch Otorhinolaryngol. (2019) 23(2):137–41. 10.1055/s-0038-1648247 30956695 PMC6449130

[49] Roll J Urban M Larson T Gailloud P Jacob P Harnsberger H. Bilateral aberrant internal carotid arteries with bilateral persistent stapedial arteries and bilateral duplicated internal carotid arteries. AJNR Am J Neuroradiol. (2003) 24(4):762–5.PMID: 12695219, PMCID: PMC8148656 12695219 PMC8148656

